# Rapid Degradation of *Caenorhabditis elegans* Proteins at Single-Cell Resolution with a Synthetic Auxin

**DOI:** 10.1534/g3.119.400781

**Published:** 2019-11-14

**Authors:** Michael A. Q. Martinez, Brian A. Kinney, Taylor N. Medwig-Kinney, Guinevere Ashley, James M. Ragle, Londen Johnson, Joseph Aguilera, Christopher M. Hammell, Jordan D. Ward, David Q. Matus

**Affiliations:** * Department of Biochemistry and Cell Biology, Stony Brook University, Stony Brook, NY 11794,; † Cold Spring Harbor Laboratory, Cold Spring Harbor, NY 11724, and; ‡ Department of Molecular, Cell, and Developmental Biology, University of California-Santa Cruz, Santa Cruz, CA 95064

**Keywords:** *C. elegans*, AID system, synthetic auxin, microfluidics, SCF complex, NHR-25

## Abstract

As developmental biologists in the age of genome editing, we now have access to an ever-increasing array of tools to manipulate endogenous gene expression. The auxin-inducible degradation system allows for spatial and temporal control of protein degradation via a hormone-inducible *Arabidopsis* F-box protein, transport inhibitor response 1 (TIR1). In the presence of auxin, TIR1 serves as a substrate-recognition component of the E3 ubiquitin ligase complex SKP1-CUL1-F-box (SCF), ubiquitinating auxin-inducible degron (AID)-tagged proteins for proteasomal degradation. Here, we optimize the *Caenorhabditis elegans* AID system by utilizing 1-naphthaleneacetic acid (NAA), an indole-free synthetic analog of the natural auxin indole-3-acetic acid (IAA). We take advantage of the photostability of NAA to demonstrate via quantitative high-resolution microscopy that rapid degradation of target proteins can be detected in single cells within 30 min of exposure. Additionally, we show that NAA works robustly in both standard growth media and physiological buffer. We also demonstrate that K-NAA, the water-soluble, potassium salt of NAA, can be combined with microfluidics for targeted protein degradation in *C. elegans* larvae. We provide insight into how the AID system functions in *C. elegans* by determining that TIR1 depends on *C. elegans* SKR-1/2, CUL-1, and RBX-1 to degrade target proteins. Finally, we present highly penetrant defects from NAA-mediated degradation of the FTZ-F1 nuclear hormone receptor, NHR-25, during *C. elegans* uterine-vulval development. Together, this work improves our use and understanding of the AID system for dissecting gene function at the single-cell level during *C. elegans* development.

*In situ* techniques for targeted protein degradation enable a detailed analysis of developmental events, mechanisms, and functions. RNAi (Qadota *et al.* 2007) and Cre or FLP-mediated recombination (Hoier *et al.* 2000; Davis *et al.* 2008; Voutev and Hubbard 2008) in *Caenorhabditis elegans* allow tissue-specific study of gene products, but the persistence of the protein of interest following RNA depletion or DNA recombination can delay manifestation of an otherwise acute phenotype. In addition, these methods are prone to off-target effects, obscuring our interpretation of experimental results (Nishimura *et al.* 2009; Hubbard 2014). Several methods have been described recently to enable tissue-specific protein degradation in *C. elegans*, including ZF1 tagging (Armenti *et al.* 2014), a GFP nanobody approach (Wang *et al.* 2017), sortase A (Wu *et al.* 2017), and auxin-mediated degradation (Zhang *et al.* 2015).

The auxin-inducible degradation system allows for rapid and conditional degradation of auxin-inducible degron (AID)-tagged proteins in *C. elegans* (Zhang *et al.* 2015) as well as in other commonly used model systems including budding yeast (Nishimura *et al.* 2009), *Drosophila* (Trost *et al.* 2016; Chen *et al.* 2018), zebrafish (Daniel *et al.* 2018), cultured mammalian cells (Nishimura *et al.* 2009; Holland *et al.* 2012; Natsume *et al.* 2016), and mouse oocytes (Camlin and Evans 2019). This protein degradation system relies on the expression of an *Arabidopsis* F-box protein called transport inhibitor response 1 (TIR1). As a substrate-recognition component of the SKP1-CUL1-F-box (SCF) E3 ubiquitin ligase complex, TIR1 carries out its function only in the presence of the hormone auxin. Once bound to auxin, TIR1 targets AID-tagged proteins for ubiquitin-dependent proteasomal degradation (Figure 1A).

**Figure 1:**
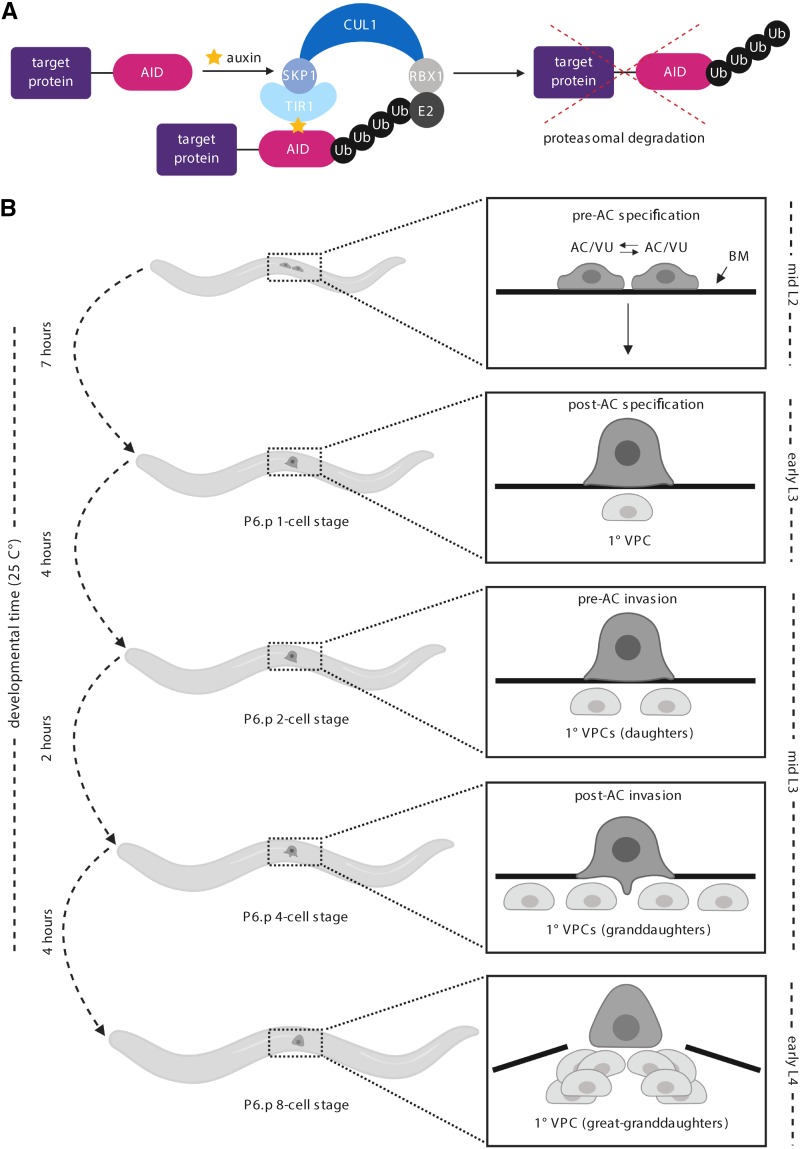
Overview of the auxin-inducible degradation system and *C. elegans* uterine-vulval development. (A) In this system, a target protein is fused to an auxin-inducible degron (AID). Heterologous expression of *Arabidopsis* TIR1 mediates robust auxin-dependent proteasomal degradation of AID-tagged proteins through the SKP1-CUL1-F-box (SCF) E3 ubiquitin ligase complex. (B) In *C. elegans*, anchor cell (AC) specification and morphogenesis of uterine-vulval attachment occurs from the mid-L2 through the early L4 stage (Schindler and Sherwood 2013). The AC is specified in a stochastic reciprocal Notch-Delta signaling event in the mid-L2 stage (top panel). Following AC specification, the AC specifies the 1° fate of the underlying vulval precursor cell (VPC) P6.p in the early L3 (second panel), which then divides three times to ultimately give rise to eight of the 22 cells of the adult vulva (bottom three panels).

The *C. elegans* AID system is robust and specific with minimal off-target effects (Zhang *et al.* 2015). However, re-evaluation of the system is needed to assess its utility among *C. elegans* researchers conducting microscopy-based single-cell biology within a narrow developmental time frame. Here, we use the indole-free synthetic auxin 1-naphthaleneacetic acid (NAA) to degrade target proteins at single-cell resolution in *C. elegans* larvae in standard growth media and physiological buffer. We also use the water-soluble, potassium salt of NAA (K-NAA) to demonstrate rapid degradation kinetics of an AID-tagged transgenic protein in a *C. elegans*-based microfluidic device for the first time (Keil *et al.* 2017). Unlike the natural auxin indole-3-acetic acid (IAA), these synthetic auxins are photostable (Yamakawa *et al.* 1979; Papagiannakis *et al.* 2017; Camlin and Evans 2019). Their use in microscopy-based experiments can prevent the production of toxic indole-derivatives during GFP excitation with blue light (Folkes and Wardman 2001; Srivastava 2002) and overcome unwanted phenotypes associated with IAA exposure (Papagiannakis *et al.* 2017; Camlin and Evans 2019). Additionally, unlike IAA (Li *et al.* 2019b), K-NAA is entirely water-soluble, bypassing the need to expose animals to low concentrations of ethanol (Zhang *et al.* 2015).

In the original description of the AID system in *C. elegans*, NHR-25 was one of the well-characterized transcription factors used to test the effectiveness of the system in the soma (Zhang *et al.* 2015). NHR-25 is the single *C. elegans* homolog of *Drosophila* FTZ-F1 and human SF-1/NR5A1 and LRH-1/NR5A2 and it regulates embryogenesis (Chen *et al.* 2004), larval molting (Asahina *et al.* 2000; Gissendanner and Sluder 2000; Frand *et al.* 2005), heterochrony (Hada *et al.* 2010), and uterine-vulval morphogenesis. During this morphogenetic event, NHR-25 works together with the HOX protein LIN-39 to regulate vulval precursor cell (VPC) differentiation (Chen *et al.* 2004). Beyond its role during vulval morphogenesis (Gissendanner and Sluder 2000; Hwang *et al.* 2007; Ward *et al.* 2013), NHR-25 also promotes specification of the uterine anchor cell (AC) in the early somatic gonad (Gissendanner and Sluder 2000; Hwang and Sternberg 2004; Asahina *et al.* 2006). Here, we utilize a newly generated GFP-AID-tagged *nhr-25* allele to visualize loss of NHR-25 in the developing somatic gonad and vulva. By exploiting the AID system’s tight temporal control of protein function, we demonstrate early on that depletion of NHR-25 results in a completely penetrant loss of AC specification, while later depletion halts VPC division. It is our hope that this synthetic auxin will be applied at all stages of *C. elegans* development, allowing for precise, rapid degradation of target proteins in a high-resolution and quantitative fashion.

## Materials and Methods

### C. elegans strains and culture conditions

Animals were maintained using standard culture conditions at 25° (Brenner 1974) and were synchronized through alkaline hypochlorite treatment of gravid adults to isolate eggs (Porta-de-la-Riva *et al.* 2012). In the main text and figure legends, we designate linkage to a promoter using a greater than symbol (>) and fusion to a protein using a double colon (::). The following alleles and transgenes were used in this study: LG I: *kry61**[nhr-23*::*AID]*; LG II: *ieSi57**[eft-3>TIR1*::*mRuby]*; LG IV: *ieSi58**[eft-3>AID*::*GFP]*, *syIs49** [**zmp-1**>GFP]*; LG X: *wrd10**[nhr-25*::*GFP*::*AID]*.

### Constructs and microinjection

SapTrap was used to construct the 30xlinker::GFP^SEC^TEV::AID degron::3xFLAG repair template (pJW1747) for generating the knock-in into the 3′ end of the *nhr-25* gene (Schwartz and Jorgensen 2016). DH10β competent *E. coli* cells, made in-house, were used for generating the plasmid. The following reagents were used to assemble the final repair template: pDD379 (backbone with F+E sgRNA), annealed oligos 3482+3483 (sgRNA), pJW1779 (5′ homology arm), 3′ homology arm PCR product, pJW1347 (30x linker for CT slot), pDD372 (GFP for FP slot), pDD363 (SEC with LoxP sites), and pJW1759 (TEV::AID degron::3xFLAG for NT slot).

The pJW1747 repair template was purified using the Invitrogen PureLink HQ Mini Plasmid DNA Purification Kit (K210001). The optional wash step in the protocol using a 4 M guanidine-HCl + 40% isopropanol solution is highly recommended, as excluding it dramatically reduced injection efficiency in our hands. N2 animals were injected with a mix consisting of 10 ng/µL of pJW1747, 50 ng/µL of pDD121 (Cas9 vector), and co-injection markers (10 ng/µL pGH8, 5 ng/µL pCFJ104, 2.5 ng/µL pCFJ90) as previously described (Frøkjær-Jensen *et al.* 2012; Dickinson *et al.* 2013, 2015). Knock-ins were isolated as previously described (Dickinson *et al.* 2015). Each knock-in junction was verified via PCR using a primer that bound outside the homology arm paired with a primer binding within pJW1747. The knock-in was backcrossed five times against wild-type N2 animals to produce JDW58. The SEC was then excised by heat-shock (Dickinson *et al.* 2015) to produce JDW59; the knock-in sequence was re-confirmed by PCR amplification and sequencing, using the oligos flanking the homology arms. JDW58 was crossed to CA1200 (*eft-3 > TIR1*::*mRuby*) to generate JDW70. The SEC was then excised (Dickinson *et al.* 2015) to produce JDW71.

pDD121, pDD363, pDD372, and pDD379 (Dickinson *et al.* 2018) were gifts from Bob Goldstein (Addgene plasmid numbers are 91833, 91829, 91824, and 91834, respectively). pJW1347 and pJW1759 will be deposited into Addgene’s repository and are also available upon request. pJW1347 and pJW1759 were generated by TOPO blunt cloning of PCR products. pJW1779 was generated by Gateway cloning into pDONR221 (Invitrogen). Oligo sequences used to generate these plasmids, the sgRNA, the 3′ homology arm, and for genotyping are in the Reagent Table.

### Auxin experiments

For all auxin experiments, synchronized L1 larval stage animals were first transferred to standard nematode growth media (NGM) agar plates seeded with *E. coli* OP50 and then transferred at the P6.p 2-cell stage (mid-L3 stage) to either OP50-seeded NGM agar plates treated with IAA, NAA, or K-NAA, or M9 buffer containing NAA in the absence of bacteria, NAA plus *E. coli* NA22, or K-NAA plus *E. coli* NA22.

For IAA and NAA experiments on plates, a 250 mM stock solution in 95% ethanol was prepared using powder IAA purchased from Alfa Aesar (A10556) and powder NAA purchased from Sigma-Aldrich (317918) and stored at -20°. IAA or NAA was diluted into the NGM agar (cooled to approximately 50°) at the time of pouring plates. Fresh OP50 was used to seed plates. For control experiments, OP50-seeded NGM agar plates containing 0.25% ethanol were used (Zhang *et al.* 2015). For K-NAA experiments on plates, a 250 mM stock solution in deionized water was prepared using powder K-NAA purchased from PhytoTechnology Laboratories (N610) and stored at 4°. For control experiments, OP50-seeded NGM agar plates were used.

Prior to each NAA experiment in M9 buffer, a fresh 1 mM (pH of 7.22) or 4 mM solution (pH of 8.14) in M9 buffer was prepared using NAA purchased in liquid form from Sigma-Aldrich (N1641). Per Sigma-Aldrich, 1 M NaOH (solvent) and water (dilutant) were used to make a 5.4 mM NAA stock solution. The pH levels described here are well within the tolerance range of *C. elegans* for pH (Khanna *et al.* 1997). M9 buffer alone (pH of 7.13) was used as a control. A detailed protocol for performing liquid-based NAA-mediated degradation experiments can be found in File S1. For experiments conducted in the microfluidic platform, a fresh solution of 4 mM NAA (derived from N1641; Sigma-Aldrich) or K-NAA (derived from N610; PhytoTechnology Laboratories) in M9 buffer containing *E. coli* NA22 was prepared and stored at 4° for up to 2 weeks. M9 buffer containing NA22 was used as a control. See File S2 for a detailed protocol describing the preparation of media for the microfluidic device.

### Brood size and viability assays

Brood size and viability assays were performed as described (Zhang *et al.* 2015). Briefly, L4 hermaphrodites were picked onto individual MYOB plates containing 0% ethanol (K-NAA control), 0.25% ethanol (IAA control), 4 mM K-NAA, or 4 mM IAA. Animals were then transferred to new plates daily over 4 days. The eggs laid on each plate were counted after removing the parent and viable progeny were quantified when the F1 reached L4 or adult stages (2-3 days post egg-laying). At this point, we also scored for dead eggs. Brood size is the sum of live progeny and dead eggs. Percent embryonic lethality was determined by dividing dead eggs by total eggs laid.

### RNAi experiments

RNAi targeting *cul-1* was constructed by cloning 997 bp of synthetic DNA based on its cDNA sequence available on WormBase (wormbase.org) into the highly efficient T444T RNAi vector (Sturm *et al.* 2018). The synthetic DNA was generated by Integrated DNA Technologies (IDT) as a gBlock gene fragment and cloned into the BglII/SalI restriction digested T444T vector using the NEBuilder HiFi DNA Assembly Master Mix (E2621). RNAi feeding strains silencing *skr-1**/2*, *skr-7*, *skr-10*, and *rbx-1* were obtained from the Vidal RNAi library (Rual *et al.* 2004).

### Scoring defects in anchor cell (AC) specification

Synchronized L1 stage *eft-3 > TIR1*::*mRuby*; *nhr-25*::*GFP*::*AID* animals were plated onto OP50 NGM agar plates containing either control or 4 mM NAA and grown for 24 hr at 25° until the early L3 stage (P6.p 1-cell stage), after the normal time of AC specification. Images were acquired as specified below to score for the presence or absence of an AC, visualized by characteristic morphology using DIC optics.

### Scoring vulval precursor cell (VPC) arrest

Synchronized L1 stage *eft-3 > TIR1*::*mRuby*; *nhr-25*::*GFP*::*AID* animals were plated onto OP50 NGM agar plates and allowed to grow until the P6.p 1-cell stage. Animals were then washed off plates with M9 and transferred onto plates containing either control or 4 mM NAA and grown at 25° until the mid-L3 stage, after the normal time of P6.p cell division. Images were acquired as specified below to score for P6.p divisions using DIC optics. Remaining animals were scored for plate level adult phenotypes approximately 24 hr later.

### Image acquisition

Images were acquired using a Hamamatsu Orca EM-CCD camera and a Borealis-modified Yokagawa CSU-10 spinning disk confocal microscope (Nobska Imaging, Inc.) with a Plan-APOCHROMAT x 100/1.4 oil DIC objective controlled by MetaMorph software (version: 7.8.12.0). Animals were anesthetized on 5% agarose pads containing 10 mM sodium azide and secured with a coverslip. Imaging on the microfluidic device was performed on a Zeiss AXIO Observer.Z7 inverted microscope using a 40X glycerol immersion objective and DIC and GFP filters controlled by ZEN software (version 2.5). Images were captured using a Hamamatsu C11440 digital camera. For scoring plate level phenotypes, images were acquired using a Moticam CMOS (Motic) camera attached to a Zeiss dissecting microscope.

### Image processing and analysis

All acquired images were processed using Fiji software (version: 2.0.0-rc-69/1.52p) (Schindelin *et al.* 2012). To quantify AC- or VPC-specific degradation of AID::GFP, images were captured at the P6.p 2-cell stage and 4-cell stage (mid-L3 stage) at time points 0, 30, 60, 90, and 120 min in the absence or presence of auxin. Expression of *eft-3>*AID::GFP was quantified by measuring the mean fluorescence intensity (MFI) of ACs and VPCs subtracted by the MFI of a background region in the image to account for camera noise. Cells were outlined using the freehand selection tool in Fiji. Data were normalized by dividing the MFI in treated or untreated animals at time points 30, 60, 90, and 120 min by the average MFI in untreated animals at 0 min. For experiments utilizing RNAi, only ACs were measured due to the variable sensitivity of VPCs to RNAi (Bourdages *et al.* 2014; Matus *et al.* 2014). To quantify AC-specific degradation of AID::GFP in animals fed RNAi overnight, images were captured at the P6.p 2-cell stage before auxin treatment and 60 min post-treatment. *eft-3 > AID*::*GFP* expression in the AC was quantified as described above. Data were normalized by dividing the MFI in auxin treated animals by the average MFI in untreated animals. To analyze NHR-25::GFP::AID degradation, GFP levels were quantified by measuring the MFI in individual GFP-expressing nuclei in the AC/VU, AC, or VPCs subtracted by the MFI of a background region in the image to account for background noise. Nuclei were outlined using the threshold tool in Fiji; for animals with no detectable GFP signal, the corresponding DIC image was utilized to identify the nucleus. Images of L3 larvae were captured in a *C. elegans* larvae-specific microfluidic device (Keil *et al.* 2017). To quantify AID::GFP degradation, animals were loaded into the microfluidic chamber and fed NA22 bacteria. Images were captured at time points 0, 30, 60, 90, and 120 min with or without auxin. Here *eft-3>AID*::*GFP* expression was quantified by measuring the MFI in whole larvae or larval pharynxes subtracted by the MFI of a background region in the image to account for background noise. Whole larvae or their pharynxes were outlined using the freehand selection tool in Fiji. Data were normalized by dividing the MFI in treated or untreated animals at time points 30, 60, 90, and 120 min by the average MFI in untreated animals at timepoints 30, 60, 90, and 120 min, respectively to account for photobleaching from imaging the same animal. Cartoons were created with BioRender (biorender.com) and ChemDraw software (version: 18.0). Graphs were generated using Prism software (version: 8.1.2). Figures were compiled using Adobe Photoshop (version: 20.0.6) and Illustrator (version: 23.0.26).

### Statistical analysis

A power analysis was performed to determine the sample size (*n*) needed per experiment to achieve a power level of 0.80 or greater (Cohen 1992; Pollard *et al.* 2019). Statistical significance was determined using either a two-tailed unpaired Student’s *t*-test or Mann Whitney *U*-test. *P* < 0.05 was considered statistically significant (Penfield *et al.* 2018). The figure legends specify when error bars represent the standard deviation (SD) or interquartile range (IQR).

### Data availability

Worm strains CA1202, CA1204, and PS3239 are available to order from the *Caenorhabditis* Genetics Center. All other strains are available upon request. The data that support the findings of this study are available upon reasonable request. Supplemental material available at figshare: https://doi.org/10.25387/g3.10277960.

## Results and Discussion

### NAA is a synthetic alternative to the natural auxin IAA

Given the recent advances in CRISPR/Cas9 genome-editing technology (Dickinson and Goldstein 2016; Dokshin *et al.* 2018), the auxin-inducible degron (AID) with or without a fluorescent reporter (*e.g.*, GFP or its derivatives) can be inserted into a genomic locus of interest (Röth *et al.* 2019). Though this technology can be applied with relative ease, there are certain limitations that exist with the use of the natural auxin indole-3-acetic acid (IAA), including its limited solubility in water (Li *et al.* 2019b). While levels of ethanol present in IAA plates (0.25–1.52%) are well below the threshold for causing a physiologic response (Morgan and Sedensky 1995; Kwon *et al.* 2004), higher percentages of ethanol (7%) have been shown to cause rapid changes in *C. elegans* gene expression (Kwon *et al.* 2004). A potentially more problematic limitation of IAA for live-cell imaging-based applications is cytotoxicity related to excitation with UV and blue light (Folkes and Wardman 2001; Srivastava 2002). Specifically, IAA has been shown in budding yeast (Papagiannakis *et al.* 2017) and mouse oocytes (Camlin and Evans 2019) to cause cytotoxicity, likely due to acceleration of the oxidative decarboxylation of IAA to methylene-oxindole (Srivastava 2002). In budding yeast, IAA exposure during live-cell imaging suppressed cell proliferation (Papagiannakis *et al.* 2017), while mammalian oocytes failed to complete meiotic maturation (Camlin and Evans 2019). In both systems, the use of the synthetic auxin, 1-naphthaleneacetic acid (NAA), rescued these cytotoxic responses. For these reasons, we chose to examine whether NAA would also function in *C. elegans* to degrade AID-tagged proteins in the presence of TIR1. Ultimately, we wished to evaluate AID-mediated degradation (Figure 1A) in single cells and tissues using live-cell imaging. Thus, we determined the kinetics of protein degradation using spinning disk confocal microscopy, rather than using low-magnification microscopy and Western blot analysis to measure protein levels in whole animals. Our rationale for choosing this approach was to avoid perturbing cells of interest in their native state, while measuring protein abundance in single cells with high spatial resolution *in vivo*. We chose to focus primarily on the L3 stage of post-embryonic development due to many of the dynamic cellular behaviors occurring over relatively short time scales (within minutes to hours) in this developmental window, including uterine-vulval attachment and vulval morphogenesis (Figure 1B) (Gupta *et al.* 2012).

**Figure 2:**
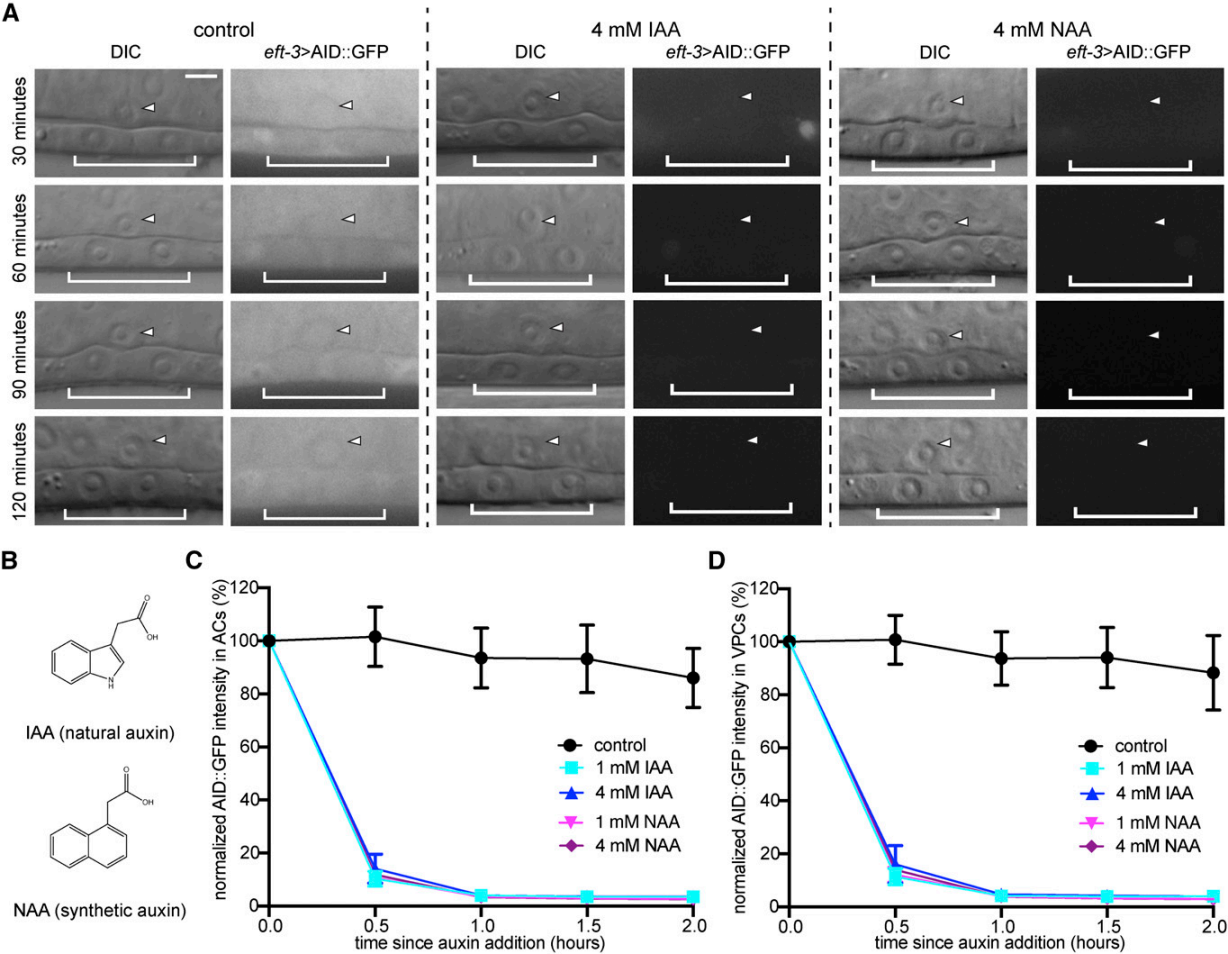
Comparison of IAA- and NAA-mediated degradation in the *C. elegans* AC and VPCs. (A) DIC and corresponding GFP images of ACs (arrowheads) and underlying 1° fated VPCs (brackets) from mid-L3 stage animals at the P6.p 2-cell stage. Animals expressing AID::GFP and TIR1::mRuby under the same *eft-3* promoter were treated with natural auxin indole-3-acetic acid (IAA) and synthetic auxin 1-naphthaleneacetic acid (NAA) in NGM agar containing OP50. (B) Chemical structure of IAA and NAA. (C, D) Rates of degradation determined by quantifying AID::GFP levels in ACs (C) and VPCs (D) following auxin treatment. Data presented as the mean± SD (*n* ≥ 30 animals examined for each time point).

To analyze the kinetics of AID-mediated degradation in the uterine anchor cell (AC) and underlying vulval precursor cells (VPCs) (Figure 1B), we utilized a previously published strain expressing AID::GFP and TIR1::mRuby under the same ubiquitously expressed *eft-3* promoter (Zhang *et al.* 2015). The single-cell abundance of AID::GFP was measured over time in mid-L3 stage animals exposed to different concentrations of auxin incorporated into standard *C. elegans* solid culture media (Figure 2A). In addition to testing the natural auxin IAA, we also tested whether it was possible to perform auxin-inducible degradation in the AC and VPCs using the synthetic auxin analog NAA (Figure 2B). In the presence of ≥ 1 mM IAA or NAA, AID::GFP abundance in the AC and VPCs was reduced by approximately 80% of its initial level within 30 min (Figure 2C-D). Within 60 min, AID::GFP was virtually undetectable (Figure 2C-D). These results indicate that NAA can serve as a viable substitute to IAA for targeted protein degradation in *C. elegans* larvae.

**Figure 3:**
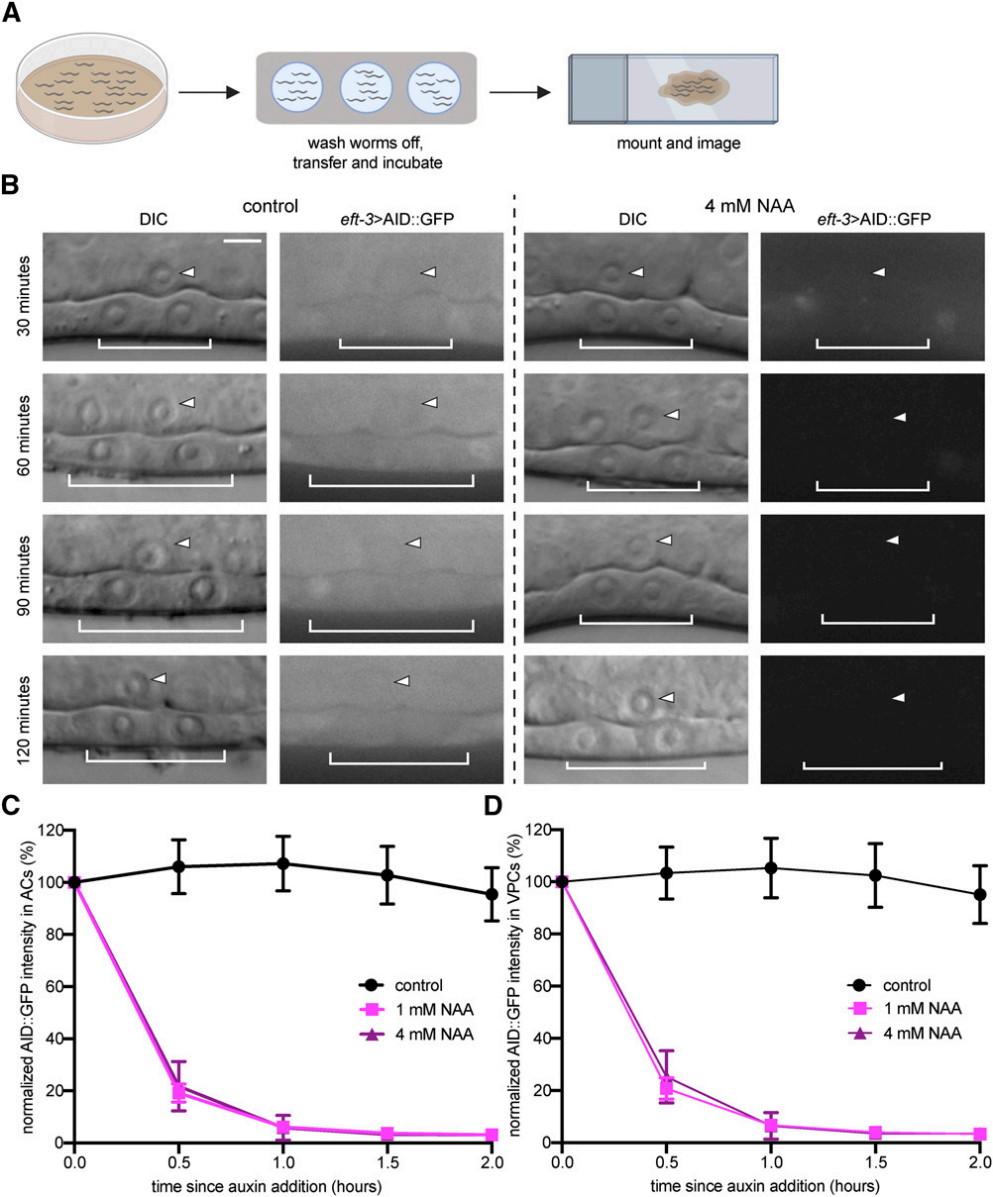
Degradation kinetics of NAA in physiological buffer. (A) Schematic representation of the liquid-based NAA-mediated degradation protocol for use in high-resolution microscopy. (B) DIC and corresponding GFP images of ACs (arrowheads) and underlying VPCs (brackets) from mid-L3 stage animals at the P6.p 2-cell stage. Animals expressing AID::GFP and TIR1::mRuby under the same *eft-3* promoter were treated with NAA in M9 buffer. (C, D) Rates of degradation were determined by quantifying AID::GFP in ACs (C) and VPCs (D) following auxin treatment. Data presented as the mean± SD (*n* ≥ 30 animals examined for each time point).

To compare degradation kinetics on plate-based growth to depletion in liquid media, we again measured AID::GFP abundance in the AC and VPCs in mid-L3 stage animals exposed to different concentrations of liquid NAA diluted in M9 buffer (Figure 3A). In the presence of ≥ 1 mM NAA, AID::GFP in the AC and VPCs was reduced by 80%, as compared to initial levels, within 30 min and was nearly undetectable within 60 min (Figure 3B-D). These results show that NAA can also induce auxin-dependent degradation in liquid media in *C. elegans*, reducing the need to rear animals on auxin plates.

### K-NAA is an option for C. elegans researchers employing microfluidics

**Figure 4:**
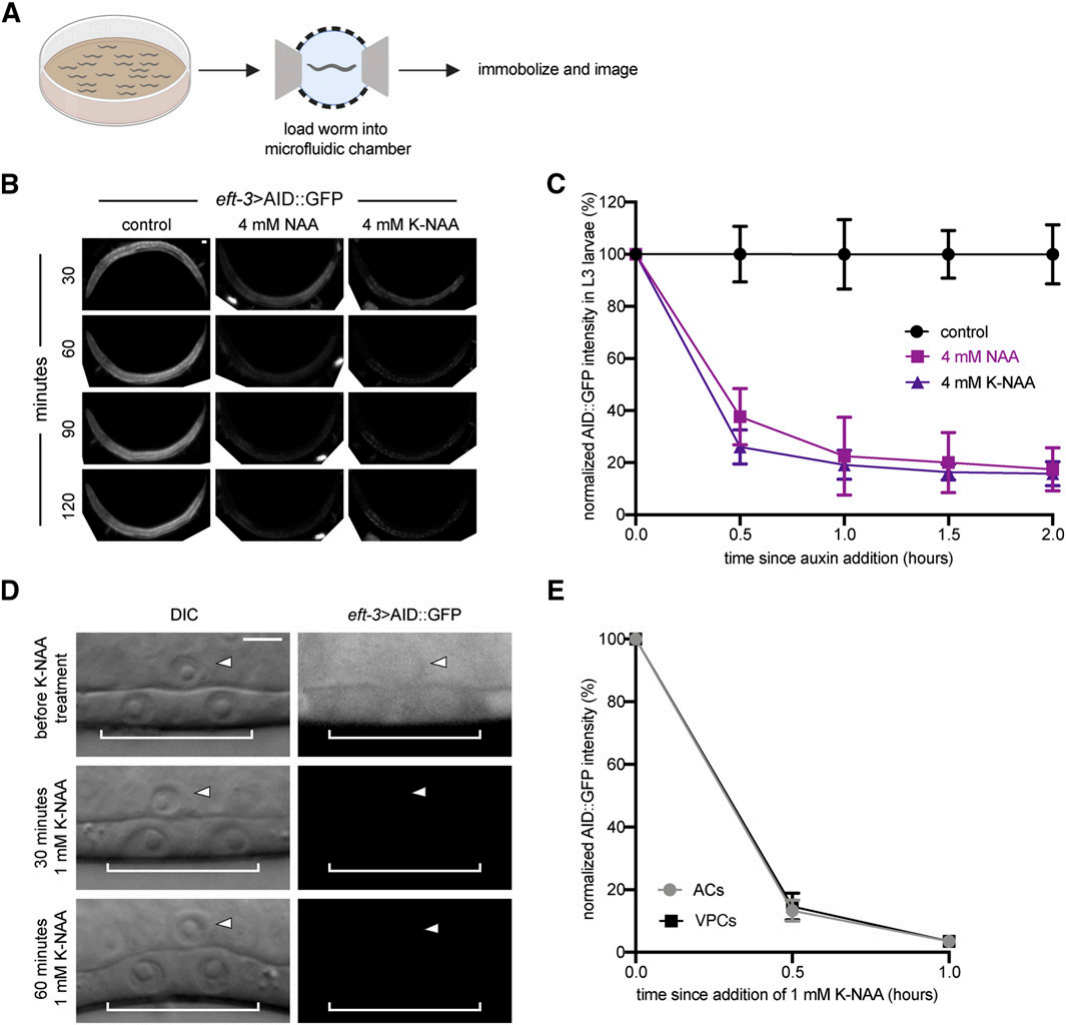
K-NAA degradation kinetics in a *C. elegans*-based microfluidic device and traditional solid growth media. (A) Schematic representation of the microfluidics-based approach (Keil *et al.* 2017). (B) Images of *eft-3>AID*::*GFP* expression from mid-L3 stage animals in control conditions (M9 buffer containing NA22 only) or conditions where a 4 mM NAA or K-NAA solution in M9 buffer containing NA22 was perfused through the microfluidic chamber for the time indicated. Anterior is left and ventral is down. (C) Rates of degradation were determined by quantifying AID::GFP in whole animals following auxin treatment. Data presented as the mean± SD (*n* ≥ 4 animals examined for each time point). (D) DIC and corresponding GFP images of ACs (arrowheads) and underlying VPCs (brackets) from mid-L3 stage animals at the P6.p 2-cell stage. Animals expressing *eft-3*>AID::GFP and *eft-3*>TIR1::mRuby were imaged before, 30 min, and 60 min after 1 mM K-NAA exposure. (E) Rates of degradation determined by quantifying AID::GFP in ACs and VPCs following K-NAA treatment. Data presented as the mean± SD (*n* ≥ 26 animals examined for each time point).

The ability to easily dilute NAA in physiological buffer (M9) raises the possibility of performing protein degradation experiments paired with microfluidics where individual animals can be imaged over long periods of time at cellular resolution. The water-soluble, potassium salt of NAA (K-NAA) serves as an attractive option for this purpose, as it has a higher concentration range (0–250 mM) than the NAA we diluted in M9 buffer (0–5.4 mM). To compare degradation kinetics between NAA and K-NAA, we time-lapsed L3 stage animals using a microfluidic device optimized for long-term imaging of *C. elegans* larvae (Keil *et al.* 2017), assessing depletion of ubiquitously expressed *AID*::*GFP* in trapped animals (Figure 4A). At the L3 stage, animals were loaded into the microfluidic chamber in M9 and flushed with a mixture of M9, 4 mM NAA or K-NAA, and *E. coli* NA22 as a bacterial food source (Keil *et al.* 2017). The animals were imaged every 30 min for 2 hr. During image acquisition, animals were temporarily immobilized by manually increasing the negative pressure on the compression layer of the device (Keil *et al.* 2017). Between timepoints, animals were allowed to move and feed freely in 4 mM NAA or K-NAA combined with NA22 in M9. Although degradation kinetics were slower than those observed in NAA in NGM plates or M9 alone, we still observed approximately 62–74% reduction of AID::GFP within the first 30 min of NAA or K-NAA exposure, and nearly 80% depletion of whole animal AID::GFP within 1 hr (Figure 4B-C). Our results may be underrepresenting the overall loss of AID::GFP as we did not account for gut autofluorescence in our quantification of fluorescence intensity in whole animals (Teuscher and Ewald 2018). Taking this into consideration, we also quantified depletion of AID::GFP in the pharynx, observing greater than 77% AID::GFP depletion within the first 30 min in larval pharynxes (Supplemental Material, Figure S1A). Thus, our results demonstrate that AID-tagged proteins can be depleted in a microfluidic platform which when combined with long-term, high-resolution imaging provides a powerful tool for studying post-embryonic *C. elegans* development at cellular resolution.

### K-NAA can be incorporated into traditional C. elegans growth media

*C. elegans* lifespan and behavioral assays can involve subtle phenotypes that are sensitive to environmental perturbations. Accordingly, the low level of ethanol present in IAA plates (Zhang *et al.* 2015) is not optimal for these types of assays. The reduced bacterial growth on high concentrations of IAA (4 mM) (Zhang *et al.* 2015) could also affect nutrition (Cabreiro *et al.* 2013), making a fully water-soluble form of auxin an attractive alternative. As such, we first wanted to determine if K-NAA also inhibited bacterial growth at high concentrations. Much to our surprise, we observed more robust *E. coli* OP50 growth on K-NAA compared to IAA or NAA with no trade-off in degradation kinetics (Figure S1B**)**. In fact, in the presence of 1 mM K-NAA the AID::GFP abundance in the AC and VPCs was almost undetectable within 60 min (Figure 4D-E). Furthermore, maintenance on K-NAA resulted in similar brood sizes compared to control, as did maintenance on IAA; however, K-NAA produced a modest but statistically significant (*P* = 0.0223) reduction in embryonic lethality compared to IAA treatment (Table S1). Consistent with this result, higher levels of toxicity were observed when using IAA over NAA in studies investigating circadian rhythm biology in *Drosophila* (Chen *et al.* 2018).

### The AID system functions through specific components of the C. elegans SCF complex to degrade target proteins

**Figure 5:**
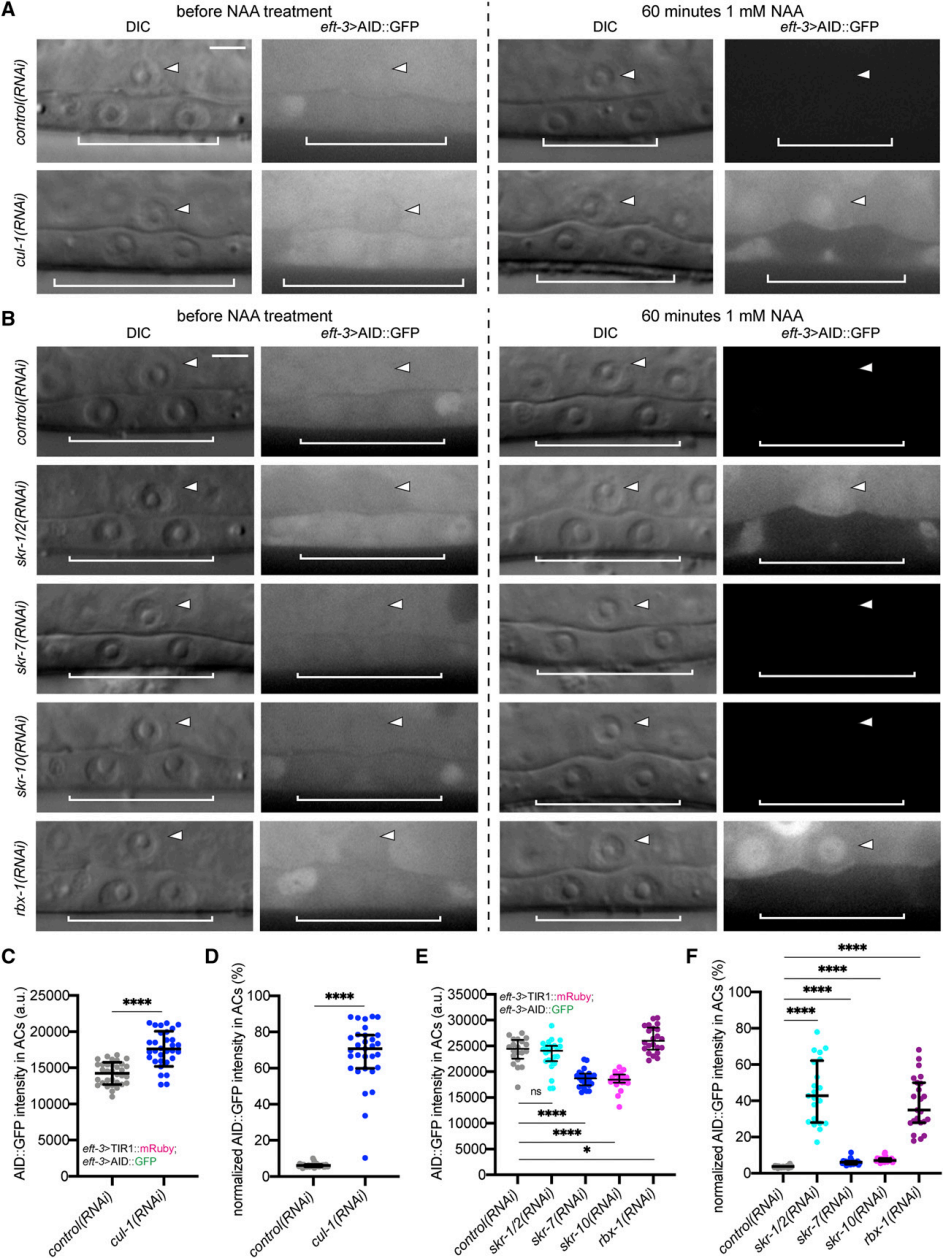
Inhibition of SCF complex member expression suppresses TIR1-dependent degradation in the *C. elegans* AC. (A, B) DIC and corresponding GFP images of ACs (arrowheads) and underlying VPCs (brackets) from mid-L3 stage animals at the P6.p 2-cell stage. Animals expressing AID::GFP and TIR1::mRuby under the same *eft-3* promoter were treated with *cul-1**(RNAi)* (A) and *skr-1**/2*, *skr-7*, *skr-10* and *rbx-1**(RNAi)* (B). (C) Quantification of AID::GFP in ACs following *cul-1**(RNAi)* treatment. Data presented as the mean± SD (*n* ≥ 30 animals examined for each, and *P* < 0.0001 by a Student’s *t*-test). (D) Quantification of AID::GFP in ACs following treatment with NAA. Data presented as the median ± IQR (*n* ≥ 30 animals examined for each, and *P* < 0.0001 by a Mann Whitney *U*-test). (E) Quantification of AID::GFP in ACs following RNAi of *skr* genes and *rbx-1*. Data presented as the median ± IQR (*n* ≥ 20 animals examined for each, and *P* values by a Mann Whitney *U*-test). *P < 0.05, ****P < 0.0001, ns not significant. (F) Quantification of AID::GFP in ACs following treatment with NAA. Data presented as the median ± IQR (*n* ≥ 21 animals examined for each, and *P* values by a Mann Whitney *U*-test). ****P < 0.0001.

As a heterologous system, researchers have shown that the *Arabidopsis* F-box protein TIR1 interacts with the *S. cerevisiae* CUL1 homolog Cdc53 (Nishimura *et al.* 2009). Whether *Arabidopsis* TIR1 also functions through *C. elegans* proteins homologous to budding yeast SCF proteins is unknown. Thus, to examine interactions between TIR1 and SCF complex proteins in *C. elegans*, we used RNAi technology directed against components of the SCF complex and quantified AID::GFP in the presence and absence of NAA (Figure 5). This experiment was designed to provide insight into the mechanism through which the AID system depletes target proteins in *C. elegans* and as an intersectional proof-of-concept test of combining auxin-based depletion with a RNAi feeding approach. Briefly, the SCF complex consists of three invariant components, SKP1, CUL1 and RBX1 and an interchangeable subunit, an F-box protein. In contrast to yeast and humans, which contain only one functional SKP1 protein, the scaffold protein CUL-1 is known to interact with eight SKP1-related (SKR) adaptor proteins in *C. elegans*, including SKR-1, -2, -3, -4, -7, -8, -9 and -10 (Nayak *et al.* 2002; Yamanaka *et al.* 2002). Of all the *SKP1*-related genes, *C. elegans **skr-1* and human *SKP1* share the greatest sequence homology (Yamanaka *et al.* 2002).

To test the role of CUL-1 in AID-mediated protein depletion, we generated a new RNAi construct targeting *cul-1* in the upgraded T444T RNAi targeting vector (Sturm *et al.* 2018). Notably, this vector contains T7 terminator sequences, which prevents non-specific RNA fragments from being synthesized from the vector backbone (Sturm *et al.* 2018). This vector modification increases the efficiency of mRNA silencing over the original L4440 vector (Sturm *et al.* 2018). We hypothesized that depleting CUL-1 would strongly interfere with the proteasomal machinery and thus protein turnover. To assess the abundance of AID::GFP in the *C. elegans* AC, we treated animals with either *control(RNAi)* or *cul-1**(RNAi)*. As before, we made use of a strain expressing AID::GFP and TIR1::mRuby from an *eft-3* driver (Zhang *et al.* 2015), and we examined animals at the P6.p 2-cell stage (Figure 5A). RNAi knockdown of *cul-1* resulted in a modest but statistically significant increase in the abundance of AID::GFP in the AC compared to *control(RNAi)* treatment (+19%, *n* = 31 and 33 animals, respectively, *P* < 0.0001) (Figure 5C). This result suggests that there is TIR1-dependent, auxin-independent depletion of AID::GFP, similar to reports in other systems (Morawska and Ulrich 2013; Natsume *et al.* 2016; Nishimura and Fukagawa 2017; Zasadzińska *et al.* 2018). To further test this notion, we assessed GFP abundance (with or without an AID tag) in the AC in animals lacking TIR1::mRuby. *AID*::*GFP* and *GFP* expression were driven by *eft-3* and *zmp-1* promoters, respectively, and we performed the experiment on animals at the P6.p 2-cell stage. We did not assess GFP abundance in the VPCs due to the variable sensitivity of this tissue to RNAi (compare Figure 5A and S2) (Bourdages *et al.* 2014; Matus *et al.* 2014). Depletion of CUL-1 via RNAi in animals expressing AID::GFP (-1.8%, *n* = 29 animals for both treatments, *P* = 0.1168) (Figure S3A-B) or GFP (-2.4%, *n* = 20 animals for both treatments, *P* = 0.7682) (Figure S3C-D) resulted in a slight decrease in AID::GFP and GFP abundance compared to treatment with *control(RNAi)*, though it was not statistically significant in either case. The modest decrease in protein abundance in animals lacking TIR1::mRuby suggests that knockdown of CUL-1 might mildly perturb protein homeostasis, but TIR1-mediated proteasomal degradation of AID-tagged proteins independent of auxin exposure requires endogenous levels of CUL-1 to function robustly.

Next, we tested whether depletion of CUL-1 would inactivate AID-mediated protein degradation. We fed synchronized L1 stage animals with RNAi targeting *cul-1*, and then treated animals at the P6.p 2-cell stage with 1 mM NAA for 60 min before quantifying AID::GFP degradation in the AC (Figure 5A). For *control(RNAi)*-treated animals, the abundance of AID::GFP in the AC was nearly undetectable within 60 min (-94%, *n* = 33 animals) (Figure 5D). In contrast, the abundance of AID::GFP in the AC was reduced by only 29% within 60 min for animals treated with *cul-1**(RNAi)* (*n* = 31 animals, *P* < 0.0001) (Figure 5D). To test whether the reduction of degradation following *cul-1**(RNAi)* is dependent on the effectiveness of RNAi-induced knockdown, we repeated the experiment using a *cul-1**(RNAi)* clone from the Vidal RNAi library. As expected for a less efficient knockdown response, AID::GFP levels were reduced by 74% within 60 min of exposure to NAA (*n* = 25 animals, *P* < 0.0001). The inefficient suppression of AID-mediated degradation in the presence of auxin highlights the increased efficiency of the *cul-1**(RNAi)* construct we built in the more effective T444T RNAi vector (Sturm *et al.* 2018). Thus, these results suggest that use of a complete loss of function of *cul-1* would fully suppress the TIR1-dependent degradation machinery.

We next wanted to determine if any of the SKR proteins in *C. elegans* function as adaptors that link CUL-1 to the F-box protein TIR1 to mediate degradation of AID-tagged target proteins. Based on the availability of RNAi clones in our Vidal library, we fed synchronized L1 stage animals with RNAi targeting four of the eight SKR adaptors known to interact with CUL-1: *skr-1*, *skr-2*, *skr-7*, and *skr-10* (Nayak *et al.* 2002; Yamanaka *et al.* 2002). Owing to the 83% sequence homology between *skr-1* and *skr-2* (likely stemming from a gene duplication event) and predicted cross-RNAi effects (Nayak *et al.* 2002), their gene names are unified in this study (hereafter referred to as *skr-1**/2*), as done previously by Nayak *et al.* (2002). We also fed animals RNAi targeting *rbx-1*, which encodes the RING finger protein in the SCF E3 ubiquitin ligase complex (Yamanaka *et al.* 2002). To assess AID::GFP abundance in the AC, we again used animals expressing *eft-3>*AID::GFP and *eft-3>*TIR1::mRuby and examined animals at the P6.p 2-cell stage (Figure 5B). RNAi of *skr-1**/2* compared to *control(RNAi)* led to differences in AID::GFP abundance that were not statistically significant (*n* = 23 and 20 animals, respectively, *P* = 0.3522). However, similar to *cul-1**(RNAi)*, RNAi silencing of *rbx-1* (*n* = 22 animals) resulted in a statistically significant increase in the abundance of AID::GFP in the AC compared to *control(RNAi)* treatment (*P* = 0.0283) (Figure 5E). Interestingly, both *skr-7**(RNAi)* (*n* = 23 animals) and *skr-10**(RNAi)* (*n* = 21 animals) resulted in a statistically significant decrease in AID::GFP abundance compared to control (*P* < 0.0001).

We also wanted to determine whether targeting *skr-1**/2*, *skr-7*, *skr-10*, and *rbx-1* could inactivate AID-mediated protein degradation. We fed synchronized L1 stage animals with RNAi targeting these SCF complex components. We treated animals at the P6.p 2-cell stage with 1 mM NAA for 60 min and quantified AID::GFP degradation in the AC (Figure 5F). For *control(RNAi)*-treated animals, the abundance of AID::GFP in the AC was once again nearly undetectable within 60 min (-96%, *n* = 21 animals) (Figure 5F). Similarly, the AID::GFP abundance in animals treated with *skr-7**(RNAi)* (*n* = 21 animals) and *skr-10**(RNAi)* (*n* = 21 animals) was undetectable within 60 min of NAA exposure (-94% and -93%, respectively). While we cannot rule out ineffective RNAi-mediated knockdown, these results suggest that neither *skr-7* nor *skr*-10 function in the SCF complex with TIR1 to mediate degradation of AID-tagged proteins. For animals treated with *skr-1**/2(RNAi)* (*n* = 21 animals, *P* < 0.0001), the abundance of AID::GFP in the AC was reduced by 57% within 60 min (Figure 5F). For animals treated with *rbx-1**(RNAi)* (*n* = 23 animals, *P* < 0.0001), the abundance of AID::GFP in the AC was reduced by 65% within 60 min (Figure 5F). These results suggest that: 1) inhibiting *cul-1*, *skr-1**/2*, or *rbx-1* expression is sufficient to suppress TIR1-dependent degradation; 2) TIR1 functions as a substrate-recognition component of the *C. elegans* CUL-1-based SCF complex, which was also previously shown in budding yeast (Nishimura *et al.* 2009); and 3) it is possible to deplete multiple targets simultaneously using both AID and RNAi technology.

Inhibiting *cul-1*, *skr-1**/2*, *or **rbx-1* expression is a valid approach for reversing AID-mediated degradation in *C. elegans*. We suggest using *cul-1*, *skr-1**/2 or **rbx-1**(RNAi)* for this purpose with caution, as they have known cell cycle-dependent functions and therefore silencing them may conflate the recovery of AID-tagged proteins with a cell cycle phenotype (Kipreos *et al.* 1996). As an alternative approach to achieving recovery of AID-tagged proteins, we propose the use of RNAi targeting *TIR1* or simply using auxinole, a commercially available inhibitor of TIR1 (Hayashi *et al.* 2012; Yesbolatova *et al.* 2019). One caveat to this approach is that auxinole is relatively expensive and thus it may be difficult to obtain stoichiometrically equivalent amounts of auxin and auxinole to truly achieve recovery of one’s protein of interest. However, for *C. elegans* researchers requiring tighter temporal control, these may be avenues worth exploring. Presently, recovery from degradation with 1 mM auxin takes up to 24 hr to fully recover expression of the target protein (Zhang *et al.* 2015). Such protein recovery kinetics are insufficient for studying events in the nematode that occur within minutes to hours such as uterine-vulval attachment, vulval morphogenesis, or many other developmental events occurring post-embryonically.

### Exploring developmental phenotypes with NAA

As our previous results demonstrate that we could effectively deplete a non-functional AID::GFP protein in the uterine AC and VPCs, we next tested whether NAA-mediated depletion of endogenous proteins could be utilized to study post-embryonic developmental events occurring over a tight temporal window. We focused on a well-studied system of organogenesis, *C. elegans* uterine-vulval cell specification and morphogenesis (Figure 1B) (Schindler and Sherwood 2013). We chose to deplete the nuclear hormone receptor, NHR-25, a homolog of *Drosophila* FTZ-F1 and human SF-1/NR5A1 and LRH-1/NR5A2. RNAi and mutant analyses have shown previously that it is initially required in the AC during the AC/VU decision for proper specification of AC fate (Gissendanner and Sluder 2000; Hwang and Sternberg 2004; Asahina *et al.* 2006) and approximately 7 hr later it is required in the underlying VPCs for cell division (Gissendanner and Sluder 2000; Chen *et al.* 2004; Hwang *et al.* 2007; Ward *et al.* 2013).

**Figure 6:**
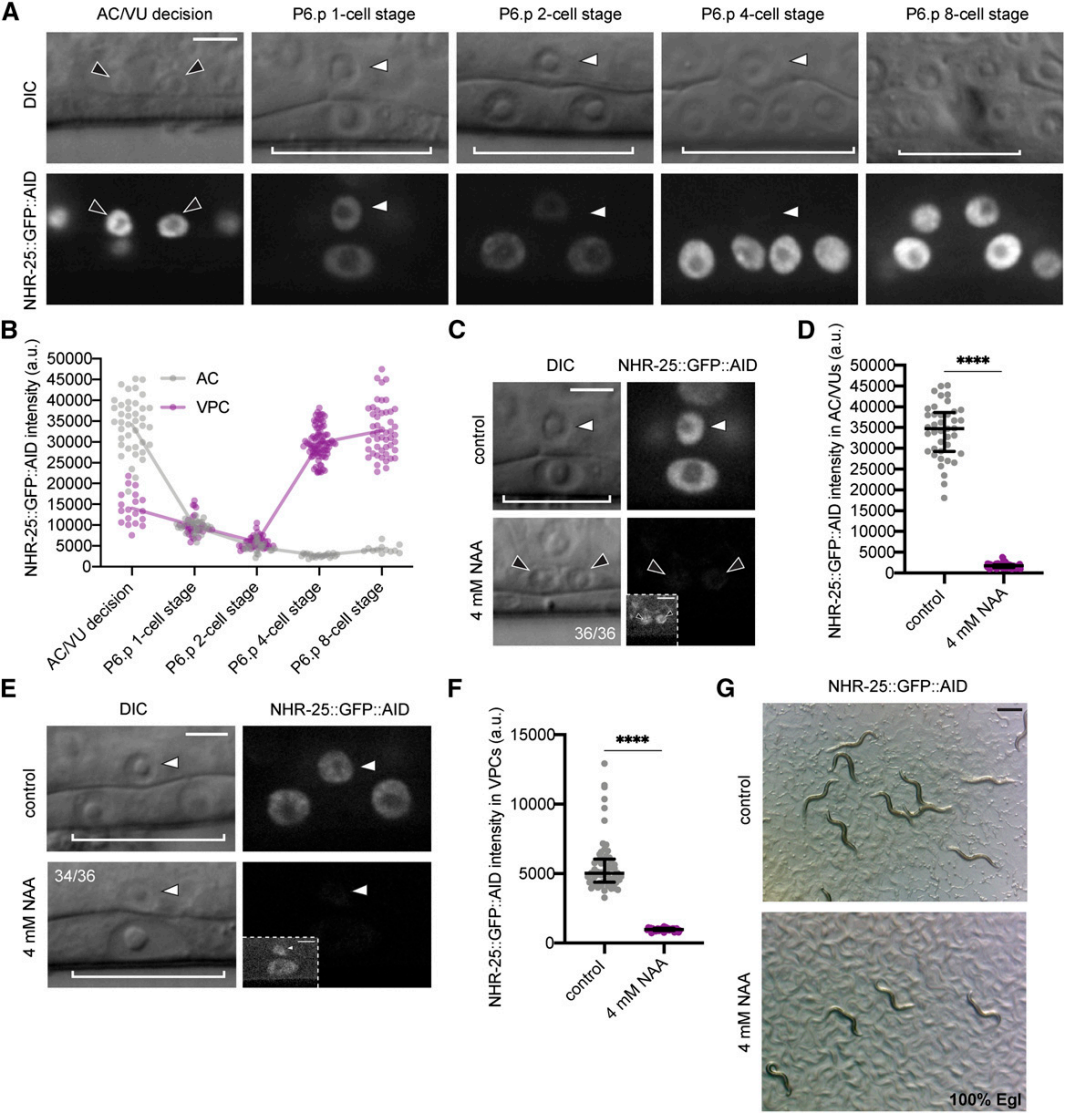
NAA-mediated degradation of NHR-25 causes AC specification and VPC division defects. (A) NHR-25::GFP::AID localizes to the nuclei of the AC/VU (black arrowheads), AC (white arrowheads) and VPCs (brackets). At P6.p 8-cell stage (far right) the AC is not in the same focal plane as the 1° VPCs. (B) Quantification of NHR-25::GFP::AID over developmental time, from the AC/VU decision to the P6.p 8-cell stage. The curve is connected by the mean at each developmental stage (*n* = 20, 31, 20, 21, and 12 animals quantified, respectively). (C) DIC and corresponding GFP images of ACs (arrowheads) and underlying VPCs (brackets) from early L3 stage animals. Animals expressing NHR-25::GFP::AID and *eft-3>*TIR1::mRuby were treated with control and 4 mM NAA. (D) Quantification of NHR-25::GFP::AID in AC/VUs following NAA treatment. Data presented as the median ± IQR (*n* ≥ 20 animals examined for each, and *P* < 0.0001 by a Mann Whitney *U*-test). (E) DIC and corresponding GFP images of ACs (arrowheads) and underlying VPCs (brackets) from mid-L3 stage animals. Animals expressing NHR-25::GFP::AID and *eft-3>*TIR1::mRuby were treated with control and 4 mM NAA. (F) Quantification of NHR-25::GFP::AID in VPCs following NAA treatment. Data presented as the median ± IQR (*n* ≥ 30 animals examined for each, and *P* < 0.0001 by a Mann Whitney *U*-test). (G) Representative images of adult plate level phenotypes following control and 4 mM NAA treatments added at the L3 stage (*n* ≥ 30 animals examined). Scale bar in (G), 500 µm.

First, we examined the *nhr-25*::*GFP*::*AID* expression pattern, and observed NHR-25::GFP::AID localization to the nuclei of the AC/VU cells during the mid-L2 stage, enrichment in the AC following specification, and nuclear localization in the 1° and 2° VPCs during all stages of vulval cell division and morphogenesis (Figure 6A). We quantified GFP fluorescence over developmental time. Consistent with previous reports based on transgene analyses (Gissendanner and Sluder 2000; Ward *et al.* 2013), endogenous *nhr-25*::*GFP*::*AID* AC expression peaks after AC specification in the early L3 at the P6.p 1-cell stage and is undetectable above background by the P6.p 4-cell stage at the time of AC invasion. Conversely, *nhr-25*::*GFP*::*AID* expression increases in the VPCs at the P6.p 4-cell stage, peaking during the morphogenetic events following AC invasion (Figure 6B). Given this temporally-driven expression pattern and based on previous experimental results from RNAi and mutant analyses (Gissendanner and Sluder 2000; Chen *et al.* 2004; Hwang and Sternberg 2004; Asahina *et al.* 2006; Hwang *et al.* 2007; Ward *et al.* 2013), we hypothesized that depleting AID-tagged NHR-25 prior to AC specification should interfere with the AC/VU decision. To test this hypothesis, we used synchronized L1 stage animals expressing *eft-3>*TIR1::mRuby and NHR-25::GFP::AID. We exposed these larvae to 4 mM NAA or control and examined animals in the early L3 stage, after the normal time of AC specification. Strikingly, all 36 animals examined showed a failure to specify the AC fate, with the presence of either one (10/36) or two (26/36) small AC/VU-like cells in the central gonad as compared to control animals (Figure 6C). Next, we repeated the experiment but waited until after AC specification, in the early L3 stage, to expose animals to control or 4 mM NAA. In all animals we detected the presence of an AC situated over P6.p, but in 34 of the 36 animals, P6.p failed to divide as compared to controls at the mid-L3 stage (Figure 6E). Quantification of NHR-25::GFP::AID abundance in AC/VU cells (Figure 6D) and VPCs (Figure 6F) demonstrated that 4 mM NAA treatment robustly depleted endogenous protein by 95% and 81%, respectively. Finally, we waited until treated animals (early L3 stage) became adults (approximately 24 hr later) and examined them for plate level phenotypes. We saw a 100% Egg-laying defect (Egl) in 4 mM NAA treated animals as compared to control treated plates (Figure 6G). Together, these results indicate that the synthetic auxin, NAA, can robustly deplete target endogenous proteins in a facile, high-throughput fashion during uterine-vulval development. This auxin should prove valuable in dissecting gene function in the future.

### Caveats of the C. elegans AID system

Several recent reports have effectively used the AID system in *C. elegans* to control protein function, including controlling spermatogenesis by manipulating SPE-44 levels (Kasimatis *et al.* 2018), depleting a mediator component to modulate longevity (Lee *et al.* 2019), examining chromosome segregation during oogenesis (Ferrandiz *et al.* 2018), examining meiotic crossover (Zhang *et al.* 2018), and revealing novel roles of neuronal gene function through conditional depletion (Serrano-Saiz *et al.* 2018). Despite the increasing frequency of AID system usage in the *C. elegans* community, there are only a handful of TIR1 driver lines published, and the importance of copy number and promoter strength has yet to be systematically assessed.

While we are optimistic that the use of the synthetic analog of auxin presented here will allow even more widespread utility of the AID system in the *C. elegans* research community, there are still some areas open to improvement for the technology. Recent reports in mammalian cell culture identified that AID-tagged proteins are depleted in an auxin-independent fashion in the presence of TIR1, relative to wild-type levels (Natsume *et al.* 2016; Li *et al.* 2019a; Sathyan *et al.* 2019). We examined if this was also occurring in *C. elegans* strains in our laboratory expressing AID-tagged proteins and TIR1. We were able to detect statistically significant auxin-independent depletion of both a ubiquitously expressed *AID*::*GFP* transgene under the *eft-3* promoter in ACs (-22%, *n* = 24 animals, *P* < 0.0001) and VPCs (-24%, *n* = 24 animals, *P* < 0.0001) (Figure S4A-B) and an endogenously tagged *nhr-25*::*GFP*::*AID* allele in ACs (-22%, *n* = 26 animals, *P* = 0.0022) and VPCs (-35%, *n* = 26 animals, *P* < 0.0001) (Figure S4C-D). As partial loss of an endogenous protein could generate a hypomorphic condition, both from the placement of the AID tag and apparent triggering of the degradation machinery, we urge caution in carefully evaluating AID-tagged alleles paired with TIR1, independent of auxin delivery. Further optimization of the AID system in *C. elegans* will hopefully ameliorate this concern, as researchers recently used the heterologous co-expression of an auxin response factor (ARF) with TIR1 to rescue auxin-independent degradation in cell culture (Sathyan *et al.* 2019).

### Conclusion

The ease of editing the *C. elegans* genome using CRISPR/Cas9 (Calarco and Friedland 2015) and the simultaneous development of heterologous gene manipulation tools is ushering in a new era of cellular and developmental biology. Several new tools available to *C. elegans* researchers require the insertion of small amino acid tags into target loci, including ZF1 tagging (Armenti *et al.* 2014), sortase A (Wu *et al.* 2017), and the AID system (Zhang *et al.* 2015). Alternatively, any GFP fusion can be targeted via a GFP nanobody tethered to ZIF1 (Wang *et al.* 2017). These genomic edits are then paired with single transgene expression to allow for targeted spatial and temporal loss-of-function approaches through manipulation of endogenous loci. Prior to their advent, spatial and temporal control of protein function was largely missing from the *C. elegans* genomic toolkit. With an ever-increasing set of these tools being optimized for *C. elegans*, it is clear that different tools will have strengths and weaknesses depending on multiple variables, including subcellular localization of target protein, availability of tissue- and cell-type specific drivers, and inducibility of depletion. Here, we optimize a powerful heterologous system, the auxin-inducible degradation system. We demonstrate that a synthetic auxin analog, NAA, and its water-soluble, potassium salt, K-NAA, can function equivalently to natural auxin. The water solubility permits easier preparation of media and allows researchers to perform experiments in liquid media and microfluidics. Importantly, the use of ethanol free K-NAA may be beneficial to *C. elegans* researchers studying behavior and aging, where introduction of ethanol may lead to confounding results. We also demonstrate the strength of the AID system for studying developmental cell biology by examining multiple spatial and temporal roles of the FTZ-F1 homolog NHR-25 during uterine and vulval morphogenesis. It is our hope that the use of the synthetic auxin NAA will complement the AID system in *C. elegans* when examining targeted protein depletion phenotypes in tissues and developmental stages of interest. As the library of tissue-specific TIR1 drivers continues to grow, we envision researchers being able to rapidly degrade proteins of interest in specific tissues and visualize the outcome at single-cell resolution.
